# Ancient DNA provides evidence of 27,000-year-old papillomavirus infection and long-term codivergence with rodents

**DOI:** 10.1093/ve/vey014

**Published:** 2018-06-15

**Authors:** Brendan B Larsen, Kenneth L Cole, Michael Worobey

**Affiliations:** 1 Department of Ecology and Evolutionary Biology, University of Arizona, 1041 E. Lowell St., Tucson, 85721 AZ, USA; 2 Northern Arizona University, School of Earth Sciences and Environmental Sustainability, 525 S. Beaver St., Flagstaff, 86011 AZ, USA

**Keywords:** paleovirology, papillomavirus, rodent, ancient DNA

## Abstract

The long-term evolutionary history of many viral lineages is poorly understood. Novel sources of ancient DNA combined with phylogenetic analyses can provide insight into the time scale of virus evolution. Here we report viral sequences from ancient North American packrat middens. We screened samples up to 27,000-years old and found evidence of papillomavirus (PV) infection in *Neotoma cinerea* (Bushy-tailed packrat). Phylogenetic analysis placed the PV sequences in a clade with other previously published PV sequences isolated from rodents. Concordance between the host and virus tree topologies along with a correlation in branch lengths suggests a shared evolutionary history between rodents and PVs. Based on host divergence times, PVs have likely been circulating in rodents for at least 17 million years. These results have implications for our understanding of PV evolution and for further research with ancient DNA from *Neotoma middens*.

## 1. Introduction

A major question in viral evolution is the timescale of coevolution between host and virus (Holmes 2009). A pattern of codivergence, or cospeciation, can occur when a host population splits and precipitates a coincident divergence in a pathogen (in this case a virus) infecting the ancestral host population (Hafner and Nadler 1988; Huyse et al. 2005). In cases where viruses are specific to a single host, the phylogeny of the virus becomes largely congruent with that of the host (Jackson and Charleston 2004; Switzer et al. 2005; Katzourakis et al. 2009; Sharp and Simmonds 2011). In contrast, if there is frequent cross-species transmission of viruses across hosts, the phylogenies will not be congruent. Determining the relative impact of these processes during viral diversification is a major question in viral macroevolution (Kitchen et al. 2011).

The *Papillomaviridae* are double-stranded circular DNA viruses that infect a broad range of vertebrates (de Villiers et al. 2004; Bernard et al. 2010; Rector and Van Ranst 2013). Papillomaviruses (PVs) are important agents of several human and animal cancers (zur Hausen 2002; Moody and Laimins 2010; Rector and Van Ranst 2013; Doorbar et al. 2015). The evolutionary history of PVs is complex, with descriptions in the literature of both strict host-virus codivergence in some taxa and cross-species transmission in others (Ong et al. 1993; Bernard 1994; Gottschling et al. 2007, 2011; Rector et al. 2007; Shah et al. 2010). PVs isolated from birds and turtles form a monophyletic group distinct to those from mammals, but within the mammalian PVs there is no strict pattern of codivergence that would unambiguously indicate an ancient relationship between host and virus. For example, PVs isolated from the same host species are often paraphyletic (García-Pérez et al. 2013, 2014). In the well-studied organism, humans, over 150 distinct PVs have been discovered (de Villiers et al. 2004). One hypothesis for this pattern is that PVs colonized new tissue types in ancestral mammals as novel environments like fur evolved (Gottschling et al. 2011). In general, PVs infecting cutaneous and epithelial tissues do not cluster together, which provides some evidence for an ancient radiation event in the primordial mammal to novel tissue types. However, this pattern is also consistent with cross-species transmission of PVs. Analysis of PV variation in humans also shows a pattern consistent with known human migration patterns out of Africa (Pimenoff et al. 2017). Thus, although it has been proposed that PVs are ancient and have been codiverging with their vertebrate hosts for millions of years, support for this hypothesis is largely lacking (Chan et al. 1992; Rector and Van Ranst 2013).

Calculating the rate of molecular evolution in viruses typically involves calibrating a molecular clock based on temporally sampled sequences (Biek et al. 2015). This approach works well for rapidly evolving RNA and DNA viruses depending on the length of sequence, sampling interval, and the number of informative sites (Firth et al. 2010). However, for slower evolving DNA viruses, matching tree topologies and relative branch lengths of host and viral phylogenies can be used to infer that codivergence has taken place, in which case host divergence times can be used to estimate rates of molecular evolution. The only published description of PV codivergence is within the mammalian family Felidae (Rector et al. 2007). Concordance between host and viral phylogenies suggests that PVs have been codiverging with their feline hosts for over 10 million years. However, direct evidence of ancient infection by PVs is still lacking.

Progress in ancient DNA techniques and novel sources of ancient DNA may allow direct confirmation of relatively ancient lineages of infectious organisms. We hypothesized that if PVs have been evolving with their hosts for a long period of time, there may be evidence of infection in some ancient DNA samples, such as middens. In general, animal middens are fossil debris piles left by various species in arid regions. These debris piles often contain fecal material, urine, plant parts, and bones. Packrat middens are made by rodents in the genus *Neotoma* (Rodentia: Cricetidae), and present an interesting opportunity to probe for ancient PV DNA. If made in a protected area, such as a dry cave or under a ledge, these middens can survive intact for tens of thousands of years (Betancourt et al. 1990).

PVs have been found in several rodent species, including the model organisms *Mus musculus* and *Rattus norvegicus*. These discoveries have opened the door to work on PV pathogenesis and to therapeutic approaches to PV infection in a model organism (Nafz et al. 2007; Joh et al. 2011; Cladel et al. 2016). A better understanding of the deep evolutionary history of rodent PVs is needed. Here, we report the presence of PV DNA in *Neotoma cinerea* midden material up to 27,000-years old. We analyzed these ancient sequence fragments with other published sequences of PVs isolated from extant rodents and demonstrate that both relative branch lengths and tree topology are congruent between the host and viral phylogenetic trees. This work thus provides both direct evidence of infection over tens of thousands of years and suggests an ancient PV-rodent relationship of at least 17 million years.

## 2. Results

### 2.1 Ancient PV sequences from rodents

We sought to test whether PV sequences could be recovered from ancient rodent samples, which would provide direct evidence of long-term infection. Sub-samples of the two ancient rodent midden samples were screened with degenerate primers to amplify a conserved region in the PV L1 gene. The middens had been aged using radiocarbon dating of their contents. The resulting ages in radiocarbon half-life years were then calibrated to a more accurate calendar year scale (Reimer and Reimer 2016; Stuiver et al. 2017). A sample of hundreds of fossil packrat fecal pellets from Chuar Valley no. 8B dated to 22,723 ± 1895 Calendar Years Ago (18,800 ± 800 C14 year BP), while a more specific date on a single fossil limber pine needle (*Pinus flexilis*) from within the midden was dated to 23,038 ± 325 Calendar Years Ago (19,132 ± 96 C14 year BP). A sample of fossil packrat fecal pellets from Chuar Valley No. 9 dated to 27,494 ± 2177 Calendar Years Ago (23,350 ± 1100 C14 year BP).

To first confirm the host identity of the rodent-origin material, we PCR-amplified and sequenced a 257 bp cytochrome *b* (cytB) sequence from one of the ancient rodent middens. It was 100% identical to *N. cinerea* by BLAST (Camacho et al. 2009). Maximum likelihood phylogenetic analyses including the PCR product and a reference panel of cytB sequences downloaded from all *Neotoma* species yielded the same results (Data not shown). No packrat DNA had ever been worked on or amplified in the lab before.

**Figure 1.**
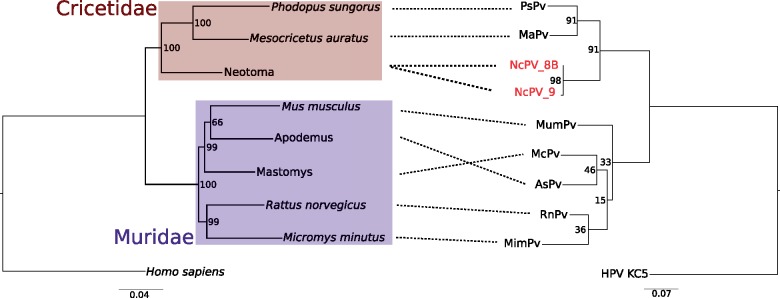
Phylogenetic congruence of PVs (right) and their hosts (left). Relationships between host and virus are indicated by dashed black lines. The host phylogeny is inferred from five loci, while the PV phylogeny is based on a 287 bp alignment of a fragment of L1 with the third codon positions stripped. Both trees were inferred with RAxML 8.2.9. Number at nodes indicates bootstrap support based on one hundred replicates. Branch lengths are in expected number of nucleotide substitutions per site. For the PV phylogeny a related human PV (GenBank accession NC_026946) was used as an outgroup. On the host phylogeny, colored boxes indicate families of rodents from the superfamily Muroidea. For three of the host species, not all loci were available on GenBank and so sequences from a related species in same genus was downloaded and used. For these cases, instead of a host species name, the genus is given.

Both midden samples were positive for PV by PCR. PVs had never been amplified in this laboratory before. The positive samples had previously been carbon dated at 27,000 and 23,000-years old (Cole 1982).These we designated ‘NcPV’ after *N. cinerea*, the host. The two ancient DNA sequences were 414- and 263-bp long. To determine the evolutionary relationship of NcPV with other previously described rodent PVs, we compared the host rodent tree (including *Neotoma*) with the PV phylogeny with the ancient NcPV sequences added (Fig. 1). The two ancient NcPV sequences, deposited ∼4,000 years apart, are distinct, differing at six nucleotide positions. NcPV clusters with other viruses isolated from rodents in the family Cricetidae with high bootstrap support (>90%). There is low bootstrap support and concordance between host and virus phylogenies for rodents in the family Muridae in these trees, possibly due to the short PV alignment.

### 2.2 Codivergence Between Rodents and PVs

**Figure 2.**
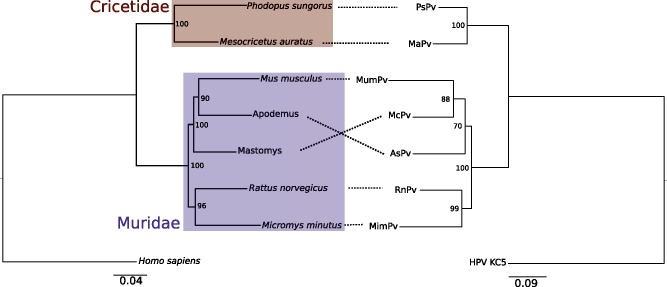
Phylogenetic congruence of papillomaviruses (right) and their hosts (left). Relationships between host and virus are indicated by spotted black lines. The host phylogeny was inferred with same loci and methods as in Figure 1, while the PV phylogeny is based on conserved regions of E1, L1, L2 with the third codon position stripped. Both trees were inferred with RAxML 8.2.9. Number at nodes indicates bootstrap support based on one hundred replicates. Branch lengths are in expected number of nucleotide substitutions per site. For the PV phylogeny a related human PV (GenBank accession NC_026946) was picked as the outgroup. On the host phylogeny, colored boxes indicate families of rodents from the superfamily Muroidea.

Since our ancient DNA data suggests a long-term relationship between rodents and PVs, we sought to more extensively test whether rodents and their associated PVs have a long-term shared evolutionary history based on similar phylogenetic patterns between hosts and viruses. Unfortunately, the short fragment we recovered from the ancient PV limited our previous analysis. Therefore, we decided to re-analyze the complete PV sequences using similar phylogenetic methods to determine if there is a pattern of codivergence between host and virus that is more robustly supported. We inferred both host and virus phylogenetic trees based on previously published PV genome sequences. First, we determined the number of independent rodent PV lineages when compared with all previously published animal PVs. There were a total of four independent rodent PV lineages (Supplementary Fig. S1). The largest clade of rodent PVs was the same lineage that contained the ancient NcPV described earlier; we focused on this clade for the codivergence analysis. The rodent PV phylogeny was inferred from an alignment of highly conserved regions of the E1, E2, L1, and L2 genes with third codon positions stripped for a total alignment length of 2,543 bp with 437 phylogenetically informative sites. The phylogenetic analysis of rodent hosts was based on one mitochondrial and four nuclear loci downloaded from GenBank, encompassing a total of 6,516 bp (including 916 phylogenetically informative sites). The resulting host phylogenetic tree recapitulates the two established rodent families, Cricetidae and Muridae, with strong support (bootstrap value = 100%; Fig. 2). Within Muridae, the genera *Rattus* and *Micromys* are highly supported as a monophyletic clade (96% bootstrap support). Finally, the clade containing *Mus*, *Apodemus*, and *Mastomys* is highly supported with bootstrap values of 100%, with the *Mus*, *Apodemus* grouping having slightly lower support value of 90%.

The rodent PV phylogeny is largely congruent with the host phylogeny, except for the placement of AsPV and McPV relative to the hosts they were isolated from—*Apodemus sylvaticus* and *Mastomys coucha*, respectively. The virus phylogeny also strongly supports the split between viruses isolated from rodents in the family Cricetidae, and rodents in the family Muridae with bootstrap values of 100% for each. Similar to the hosts, the lowest support values are found with the relationship between PVs isolated from *Apodemus*, *Mus*, and *Mastomys*, which have bootstrap support values of 70 and 88%.

**Figure 3.**
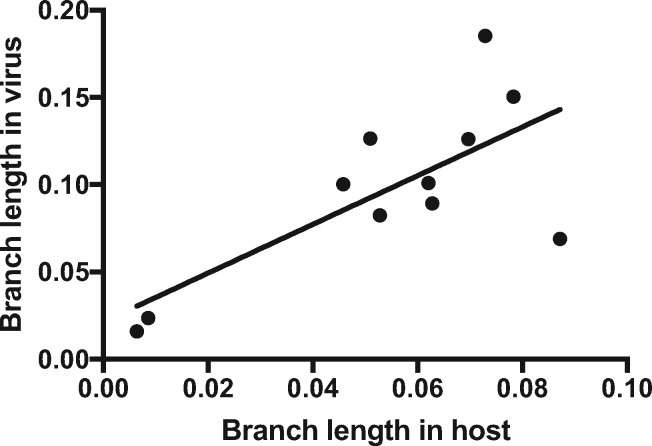
Correlation of PV branch lengths and their associated rodent hosts. Branch lengths are in expected number of nucleotide substitutions per site. Branch lengths are derived from the ML phylogeny in Figure 2. Since there is not perfect concordance between host and virus phylogenies for the *Mus, Mastomys, and Apodemus* taxa, the branch lengths to the common ancestor for the three taxa was used as a proxy instead of the individual branches.

Finally, we tested whether there is a relationship between the branch lengths of the congruent host and virus phylogenies. Branch lengths between PVs and their rodent hosts are significantly correlated, suggesting rodents and their PVs share a similar evolutionary history (*R*^2^ = 0.52, P = 0.012; Fig. 3). This pattern is expected only if host and virus have a shared evolutionary history.

**Figure 4.**
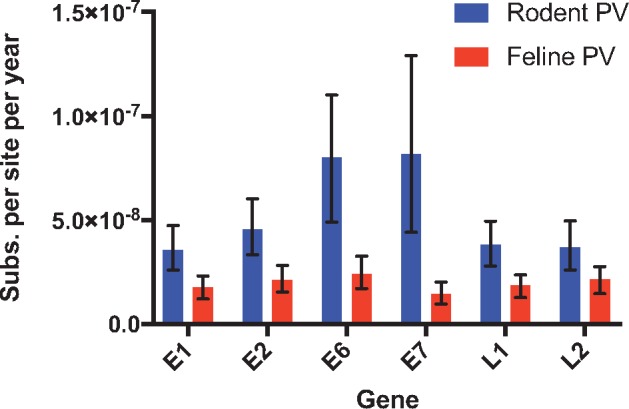
Mean estimates of rodent PV substitution rates compared with previously estimated rates of evolution in feline PVs across six genes. Bars represent 95% CIs. Estimates were based on a relaxed lognormal clock model implemented in BEAST. Calibration was based on previously reported estimates of host divergence times.

The pattern of codivergence between rodents and their PVs strongly suggests a long-term association, which justifies the calibration of a molecular clock based on host divergence dates. Based on previously published estimates of host divergence times, we estimated the substitution rates using a Bayesian relaxed clock model for rodent PVs and compared them to previously published estimates of feline PV substitution rates. The mean age estimate for the root of this clade of rodent PVs was 17.7 million years ago (95% CI 15.6–19.8mya). The mean substitution rate estimates for each gene, along with the 95% CIs intervals are shown in Fig. 4. The overall substitution rate for E1, E2, E6, E7, L1, and L2 for rodent PVs was 5.2 × 10^−8^. On average, rodent PVs had substitution rates estimated to be ∼2.8 times higher than feline PVs. The genes E6 and E7 from rodent PVs showed higher substitution rates than any of the feline PV genes.

## 3. Methods

### 3.1 DNA extraction of *Neotoma* midden material

The packrat middens analyzed for this study were originally collected in 1979 from a cliff at 1770 m of elevation on the south arm of Poston Butte, just east of Chuar Valley in the Grand Canyon, Arizona (36° 10’ 27” N.; 111° 54’ 5” W) (Cole 1990). They were part of a study that first described the elevational movement of vegetation zones within the Grand Canyon between the Pleistocene and Holocene (Cole 1982). These middens had been preserved in a repository at Northern Arizona University containing several thousand similar deposits from western North America and the Middle East. Because of the vegetation surrounding the deposits at that time, and the inclusion of fossil tooth (RM^3^) identified as being from *Neotoma* cf. *cinerea* (Cole and Mead 1981), it is most likely that the packrat species producing the middens was *N. cinerea* (bushytailed packrat), although another tooth (LM_3_) was identified as *Neotoma lepida* (Desert Packrat).

All DNA extractions and reagent preparation were performed in rooms physically separated from any PCR amplification, and the actual DNA extractions were performed in a biosafety cabinet inside of a BSL-2 laboratory. Extraction of rodent samples or PVs had never been performed in the laboratory prior, and rigorous protocols were followed to prevent contamination. During each DNA extraction, a negative control was run alongside the others to ensure that contaminants were not introduced through reagents or other sources. (None of the negative controls yielded PV amplification products.) After cutting up ∼2–5 g of bulk, unwashed midden material containing fecal pellets, samples were incubated for 18 h at 30°C with gentle agitation in 10 ml of ‘Bulat extraction buffer’ which contained the following final concentrations (adapted from Haile 2011): 0.02 g/ml Sarcosyl, 50 mM Tris-HCL pH 8.0, 20 mM sodium cloride (NaCl), 3.5% 2-mercaptoethanol, 10 mM dithiothreitol, 100 mM N-phenacylthiazolium bromide, 0.8 mg/ml proteinase K, and 50 mM of ethylenediaminetetraacetic acid (EDTA). At the end of the incubation, the temperature was increased to 56°C for 2 h. Tubes were then spun at 5,000 g for 5 min and the supernatant was added to 40 ml of binding buffer (procedure adapted from Rohland and Hofreiter 2007), 5 M guanidinium thiocyanate, 25 mM NaCl, and 50 mM Tris-HCL). Next, a suspension of 100 μl of silica was added and finally the pH was adjusted to ∼4.0 with 30% HCL. Samples were incubated for 2 h at room temperature and then spun down at 5,000 rpm for 2 min. The silica pellet was washed twice with a washing buffer, which contained a final concentration of 50% EtOH, 125 mM NaCl, 10 mM Tris-HCL, and 1 mM EDTA. Finally, the silica pellet was allowed to dry and 150 μl of molecular grade H_2_0 was added for a 10-min incubation period. This solution was then centrifuged at 8,000 rpm for 2 min to generate the DNA extraction product. As a final purification step, each sample was run through an OneStep PCR Inhibitor Removal column (Zymo Research).

### 3.2 PCR amplification of PV and *cytB* fragments from midden DNA

Primers specific for rodent PVs and *Neotoma* cytB were designed from alignments created in house by using sequences downloaded from GenBank. Negative PCR controls were included during all PCR steps and yielded no spurious amplification products. DNA was amplified using two rounds of nested PCR. In the first round, 2 μl of the DNA extraction was amplified using AmpliTaq Gold 360 DNA polymerase (Thermo Fisher Scientific), in a 50 μl reaction volume, with a final concentration of 0.4 μM of each primer, 200 μM dNTP, 1× AmpliTaq Gold 360 Buffer, and 2.5 μM of MgCl_2_. The primers used were either Pap_F1 (5'-ATYGAGGATGGDGAYATGKGTGA-3') or Pap_F0 (5'-CYWYDGGIGAGCAYTGG-3'), along with Pap_long_R_alt (5'-AACAGCTGACCATCACT-3') with the following cycling conditions: initial denaturation at 95°C for 4 min, 60 cycles of (95°C for 20 s, 50°C for 30 s, and 72°C for 30 s) and final elongation at 72°C for 5 min. In the second round of PCR, 1 μl from the first round was added to a 50 μl reaction mixture containing NEB *Taq* DNA polymerase (New England Biolabs) with a final concentration of 1 μM of each primer, 200 μM dNTP, 1× Standard *Taq* Reaction Buffer, an additional 1 μM MgCl_2_, and 1.25 units DNA polymerase using a hemi-nested set of primers, with either Pap-F0 or Pap-F2 (5'-YATWGGCTTTGGSAATATGRAYTTCA-3') and Pap_long_R_alt or Pap_long_R (5'-CCACTRGGDGTTCCAAAGTA-3'), with the following cycling conditions: initial denaturation at 95° for 2 min, 35 cycles of (95°C for 30 s, 50°C for 30 s, and 68°C for 30 s) and a final elongation at 68°C for 5 min. For the host *cytB* amplification, identical PCR conditions were used as above, except with the following primers for the first round of PCR: *Neotoma*_cytB_F1 (5'-TATTYTTYCCWGAYATCCTIGGRG-3') and *Neotoma*_cytB_R (5'-CGTAGRATTGCGTARGCRAAYAGRAA -3'). For the second round of PCR the following primers were used: *Neotoma*_cytB_F2 (5'-CCVGAYAACTAYACCCCIGCAAAYCC-3') and *Neotoma*_cytB_R. The *cytB* sequence and PV sequences are deposited into GenBank under accession numbers [X]. The PV phylogeny with the NcPV sequences added is based on a 287 bp alignment of a fragment of L1 with the third codon positions stripped.

### 3.3 Phylogenetic analysis of PV/rodent associations

A global analysis of animal PVs was performed to infer the relationship of all described isolated rodent PVs. Gene sequences were downloaded from NCBI (accession numbers available in Supplementary Table S1, and only the most conserved regions of L1, L2, and E1 were used to infer the phylogeny. Due to low sequence identity, nucleotide sequences were translated to amino acids, and a phylogeny was inferred using the LG substitution model with a gamma rate variation distribution in SeaView 4.5.3 using PhyML (Le and Gascuel 2008; Gouy et al. 2010; Guindon et al. 2010). Branch support was calculated with aLRT (Anisimova and Gascuel 2006). The phylogeny of the eight rodent hosts was inferred from publically available sequences downloaded from NCBI GenBank and included four nuclear loci (breast cancer 1 (BRCA1), Growth hormone receptor (GHR), Retinol-binding protein 3 (IRBP), and Recombination activating gene 1 (RAG-1) and one mitochondrial locus (cytB). In a few instances, sequence data were not available for a particular rodent species. For these cases, a sequence from the same genus was used. These cases are noted in the Supplementary Table S1. Sequences were aligned and trimmed in Geneious 8.1.5 to remove positions in the sequence that contained an ‘N’ (Kearse et al. 2012). Host loci were analyzed as separate partitions to infer a single species tree in RAxML 8.2.9 with the GTRGAMMA model of sequence evolution and 100 bp iterations (Stamatakis 2014).

For the codivergence analysis, rodent PV sequences included in the analysis were downloaded from GenBank, which had been isolated from *R. norvegicus* (GQ180114) (Schulz et al. 2009), *Micromys minutus* (NC_008582) (Van Doorslaer et al. 2007), *A. sylvaticus* (NC_024893) (Schulz et al. 2012), *M. musculus* (NC_014326) (Joh et al. 2011), *M. coucha* (NC_008519) (Amtmann et al. 1984), *Phodopus sungorus* (HG939559) (Kocjan et al. 2014), and *Mesocricetus auratus* (NC_022647). Alignments of each coding region were made with MUSCLE, and were then hand aligned to be in-frame in Geneious 8.2.9 (Edgar 2004). To infer the PV tree only the genes E1, E2, L1, and L2 were used due to their relative conservation compared with other genes. Phylogenetically informative segments of the alignment were extracted using GBlocks 0.91 bp with default settings keeping codons intact (Castresana 2000). Due to high saturation at the third codon position as determined by DAMBE 6.4.2 (ICC > ICC.c), third codon positions were stripped from the alignment (Xia 2013). The PV phylogeny was inferred by RAxML 8.2.9 with the GTRGAMMA model of sequence evolution and 100 bp iterations.

Substitution rates of rodent PVs were calculated with BEAST v1.8.1 (Drummond et al. 2006, 2012). Substitution rates were calculated for each gene as a separate partition for the PV genes E1, E2, E6, E7, L1, and L2. To determine the most appropriate evolutionary model all gene-specific alignments were tested in jModelTest (Posada 2008). Of the models tested, the GTR substitution model had the best fit for each alignment and was implemented with estimated base frequencies and a gamma distributed site heterogeneity model. Three calibration dates were used for the PV phylogeny: the time of the most recent common ancestors (TMRCAs) of (1) the rodent subfamily Murinae, (2) the family Cricetidae, and (3) of *Mus* plus *Mastomys*, based on previous work that incorporated fossil data to estimate divergence times of different rodent groups (Steppan et al. 2004). Prior probabilities for the TMRCA of the rodent subfamily Murinae were drawn from a normal distribution with a mean of 10.3 million years ago (mya) and a SD of 0.2 mya. For Cricetidae the mean was set to 13.8 mya and SD to 0.69 mya. And for the *Mus*/*Mastomys* ancestor, the mean was set to 8.8 mya and SD to 0.3 mya. All other priors were kept at their default settings except the ucld.mean parameter, which was changed to have a gamma distribution with a shape value of 0.01 and a scale of 100. Two independent runs were set-up with 100 million generations sampling the chains every 2000 generations. Log files were examined in Tracer v1.6 to check that ESS values were >200 and to check convergence of the two runs, with 10 million generations discarded as burnin.

## 4. Discussion

In this article, we describe ancient PV sequences derived from rodent middens, and compare them to other published rodent PVs. We show there is a correspondence in the overall tree topology and relative branch lengths for this lineage of rodent PVs and their hosts, which suggests a long-term shared evolutionary history of codivergence. Although our overall PV phylogeny reveals four independent rodent PV lineages, which does not indicate strict host/pathogen codivergence in the overall PV phylogeny, this particular clade of rodent PVs does fit a pattern of codivergence. Others have noted a pattern of codivergence in some rodent PVs (Schulz et al. 2009). However, this prior work involved fewer lineages, did not compare the differences in branch lengths between host and PVs, and did not use independent host divergence times to estimate a substitution rate. Moreover, we used ancient DNA to provide direct evidence of infection from 27,000 years before present.

One caveat with our analysis is that although a significant relationship exists between the branch lengths of the host and virus phylogenies, and overall there is high concordance between the tree topologies, there is not perfect concordance. Additionally, due to the low number of nodes we did not perform a statistical test of topological concordance between the host and virus phylogenies. Discordance between host and virus trees can arise for a number of reasons, including cross-species transmission, problems in phylogenetic reconstruction, and recombination (Huyse et al. 2005; Nieberding and Olivieri 2007). Our analysis of host relationships among rodents is based on five loci (four nuclear and one mitochondrial), which limits the power to make conclusions regarding the placement of taxa. Additionally, in some cases some loci were not available for certain taxa, so sequences from a closely related taxon were used instead. This may have introduced some noise into the estimates of the host branch lengths (but is not expected to have systematically biased the overall results.) Within Muridae, the lowest support for both the host and virus taxa is within the placement of *Mus*, *Apodemus*, and *Mastomys*. These three species were a part of an rapid radiation that occurred at the basal position of the core *Murines*, which makes phylogenetic reconstruction difficult (Steppan et al. 2004). Interestingly, phylogenetic studies involving more taxa, more loci, and a supertree approach consistently placed *Mus* and *Mastomys* as sister groups, with *Apodemus* as the outgroup, although in all cases the nodes were poorly supported, demonstrating the difficulty in resolving this rapid radiation (Steppan et al. 2005; Rowe et al. 2008). This placement of taxa differs from our host tree, but if correct would make our PV phylogeny in Fig. 2 completely congruent with the host phylogeny.

Another caveat is the limited number of sequences from this clade of rodent PVs. In total there are eight described sequences in this group isolated from rodents; however, there are over 1,300 species in the superfamily Muroidea (Michaux et al. 2001). Thus, our conclusions on codivergence in this group are based on <1% of all rodent species. Greater sampling of divergent rodent lineages for novel PVs is needed in the future.

Phylogenies of rapidly evolving viruses have been shown to recapitulate host movement and population structure over short periods of time (Biek et al. 2006; Thapa et al. 2016). In this paper we add to the building evidence that diversification patterns in relatively slowly evolving DNA virus such as PV can also match host divergences over millions of years. This long-term association has been observed in other DNA viruses. For example, DNA viruses from the family *Polyomaviridae* have likely been codiverging with their hosts for ∼500 million years (Buck et al. 2016), *Hepadnaviridae* for ∼430 million years (Lauber et al. 2017), and *Baculoviridae* for ∼310 million years (Thézé et al. 2011). Our description here of a case of apparent codivergence between some rodent groups and their PVs, along with previous evidence of codivergence of PVs in felines (Rector et al. 2007), bolsters the evidence of an ancient origin of PVs. Although the full picture is still far from clear, we can speculate about some major patterns: In mammals there are two major PV lineages that infect either mucosal or cutaneous sites. Therefore, at the base of the mammalian PV phylogeny there may have been an expansion in cellular tropism which led to the paraphyly of some groups of PVs observed today (de Villiers et al. 2004). Although the overall tree contains obvious exceptions to strict host/virus codivergence, the overall pattern of turtle and bird PVs being a distinct clade from mammalian ones suggests that PVs codiverged with the split of mammals from the turtle/crocodile/bird lineage, which would have happened ∼300 million years ago (Kumar and Hedges 1998). Finally, the recent discovery of a novel PV in a fish may push back the time of emergence even further (López-Bueno et al. 2016). Greater sampling of fish PVs and discovery of amphibian PVs are needed to better understand these deeper evolutionary patterns between host and PVs.

In this study we utilized ancient DNA to make inferences about the evolutionary history of PVs. Ancient DNA has provided powerful insights into a broad range of evolutionary and population level processes in an extensive range of organisms (Slatkin and Racimo 2016). Despite these advances, recovery of ancient viral DNA or RNA is fairly limited, with only a few studies published to date. Some examples of ancient viral nucleic acid that have been reported in the literature are phage viral DNA from a 14th century human coprolite (Appelt et al. 2014), Barley Stripe Mosaic Virus RNA recovered from dried barley grain that was ∼750 years old (Smith et al. 2015), tomato mosaic tobamovirus RNA in Greenland ice up to 140,000-years old (Castello et al. 1999) and 30,000 and 700-year-old DNA and RNA viral nucleic acid recovered from frozen Siberian ice (Legendre et al. 2014; Ng et al. 2014). The technical challenge of ensuring sequences without contamination and the paucity of suitable samples containing intact ancient viral nucleic acid likely limits these types of studies. Despite these challenges, ancient viral nucleic acid can provide insights into the timing of viral evolution (Harkins and Stone 2015).

Estimates of PV substitution rate have been fairly consistent despite differences in estimation methods and taxa analyzed, but these rodent PV rates are somewhat higher. For example, comparing PV sequences isolated from cats and dogs and using the split of these host lineages led to an estimate of 0.73–0.96 × 10^−8^ substitutions per base per year (Tachezy et al. 2002). An analysis of feline PVs yielded an estimate of 1.95 × 10^−8^ (Rector et al. 2007). We estimated an average substitution rate for 6 genes in rodent PVs of 5.2 × 10^−8^. Our estimate of the root of the rodent PV phylogeny was 17.7 million years. This represents the TMRCA of Muridae and Cricetidae and is younger than the 24.7 million years estimated from host phylogenies and the fossil record (Michaux et al. 2001; Steppan et al. 2004). Systematic underestimation of substitution rates in deep portions of viral phylogenetic trees has been noted in several viral lineages, leading to underestimation of TMRCAs (Ho et al. 2007; Wertheim and Kosakovsky Pond 2011). Accordingly, it is not surprising that our analysis also underestimated the age of the deepest node, albeit only slightly.

Our ancient PV sequences are novel for two reasons. First, although a large number of ancient viruses have been described, this represents the oldest PV sequence that we are aware of. Second, this is the first description of ancient viral DNA from an animal midden. Ancient rodent material has been used, but never middens made specifically by rodents in the genus *Neotoma* (Kuch et al. 2002; Murray et al. 2012). The fact we were able to get relatively long continuous stretches of DNA (414 bp) from a 27,000-year-old sample is somewhat surprising. Typically, DNA is thought to be best preserved at cold temperatures. In our case, middens were preserved in dry caves within the Grand Canyon. Ancient *Neotoma* middens are hard dense structures of dried urine, plant material, and fecal pellets. There may be something specific about the biochemical properties of *Neotoma* middens that enable long-term preservation of relatively long stretches of DNA. Hundreds of middens spanning from the present to over 45,000-years old have been collected and studied for plant macrofossils across western North America. Since they contain host and pathogen DNA, and likely plant DNA, they represent very promising resources to study genetic changes over tens of thousands of years across different environments in western North America, and clearly can be an important window into not just hosts but also viral pathogens.

## Supplementary Material

Supplementary Table

Supplementary Figure

## Data Availability

Sequences were deposited into Genbank with accession numbers MH136585-MH136587. **Conflict of interest:** None declared.
