# Supplementary material for: Ancient DNA provides evidence of 27,000-year-old papillomavirus infection and long-term codivergence with rodents
Source: Virus Evol. 2018 Jun 15;4(1):vey014. doi: 10.1093/ve/vey014 (PMC6007503; doi:10.1093/ve/vey014)
Supplement: Supplementary Figure [file vey014_supp_sf.docx]

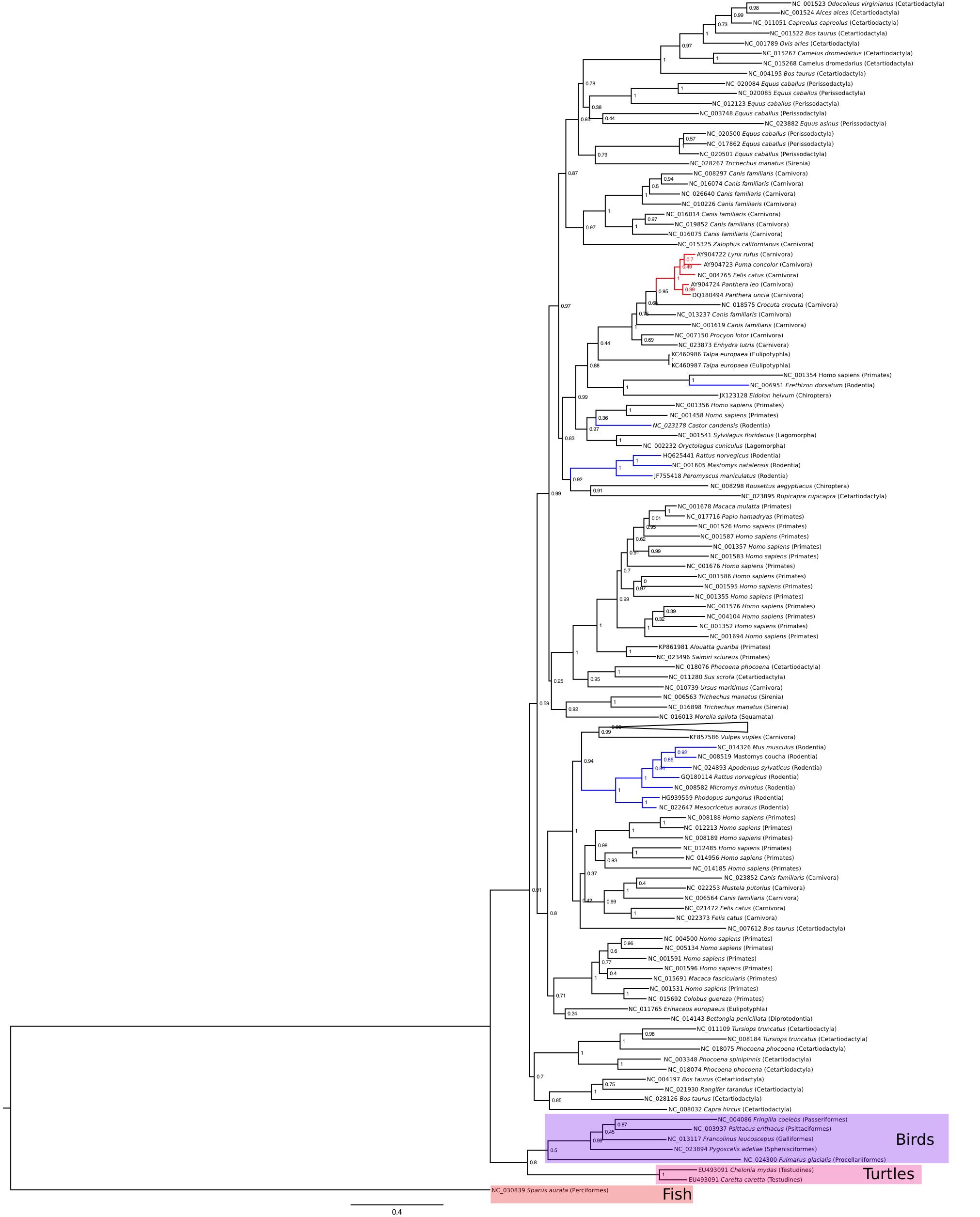


Supplementary Figure S1 **Phylogenetic relationship of all papillomavirus genomes isolated from vertebrates.** A Maximum Likelihood phylogeny was inferred based on an alignment of conserved 688 amino acids from the E1, L1, and L2 genes. Non-mammal PVs are highlighted in color. Support values at each node are derived from aLRT estimates rather than bootstrap values. Branches leading to rodent PVs are colored in blue. Branches leading to PVs isolated from Felidae are shown in red. Accession numbers of the viral sequences in genbank are given at the start of each taxa name, followed by the scientific name of the host from which the PV was isolated. Finally, the mammalian order or suborder was given at the end of each taxa name. In order to reduce the number of taxa shown, a large cluster of human-only PVs was collapsed as shown by a black triangle.
